# Basophil activation test is food adverse reactions

**DOI:** 10.1186/2045-7022-1-S1-P92

**Published:** 2011-08-12

**Authors:** Patrizia Pignatti, Giselda Colombo, Mona-Rita Yacoub, Gianni Palai, Gianna Moscato

**Affiliations:** 1 Fondazione Salvatore Maugeri, Allergy and Immunology Unit, Pavia, Italy; 2 San Raffaele Scientific Institute, IRCCS, Allergy and Immunology Unit, Milan, Italy

## Background

Some subjects with reported food adverse reactions have negative skin prick tests (SPTs) and serum specific IgE (sIgE) for the suspected foods. The aim of the present study was to compare data of basophil activation test (BAT) with SPTs and sIgE results in subjects with food adverse reactions.

## Methods

83 subjects (66/17 females/males) with reported food adverse reactions were included in the study. Eighteen atopic/allergic subjects were included as controls for the foods to which they had reported no reactions. BAT was performed on heparinazed blood incubated with food extracts. CD63 activation marker was evaluated on basophils by flow-cytometry. The stimulation index (SI) was calculated as ratio: CD63% on basophils incubated with food extract/CD63% on basophils with wash buffer.

## Results

CD63>5% and SI>2 was chosen as positive cut-off on the basis of control subjects' results. We performed 664 BATs in the subjects considered. 62,2% of the subjects presented a positive BAT for at least one food extract, 39.0% a positive SPT and 34.7% had detectable sIgE for foods. 78,2% was the concordance between BAT and SPT and 74,2% between BAT and sIgE. A positive BAT was more frequent with peanut, peach and apple extracts and was found in 91,7% of the subjects with a history of food anaphylaxis, 52,6% with urticaria/angioedema, 45,4% with gastrointestinal symptoms and 55,5% of the subjects with mixed symptoms (urticaria/angioedema and gastrointestinal symptoms). Among the subjects with a positive BAT, 20,4% had negative sIgE and 17,8% had negative SPTs.

## Conclusions

a good correlation between positive BAT and food adverse reactions was shown in subjects with a history of anaphylaxis or with mixed symptoms of food allergy. We identified a group of patients with negative in vitro and/or in vivo tests and positive BAT. In these subjects double-blind placebo control challenges should be performed to confirm their allergic condition.

